# Combined Antigen-Specific Interferon-γ and Interleukin-2 Release Assay (FluoroSpot) for the Diagnosis of *Mycobacterium tuberculosis* Infection

**DOI:** 10.1371/journal.pone.0120006

**Published:** 2015-03-18

**Authors:** Dumitru Chesov, Christoph Lange, Franziska Daduna, Valeriu Crudu, Rosemarie Preyer, Martin Ernst, Barbara Kalsdorf

**Affiliations:** 1 Division of Pneumology and Allergology, State University of Medicine and Pharmacy "Nicolae Testemitanu", Chisinau, Republic of Moldova; 2 Division of Clinical Infectious Diseases, Research Center Borstel, Borstel, Germany; 3 German Center for Infection Research, Tuberculosis Unit, Borstel, Germany; 4 International Health/Infectious Diseases, University of Lübeck, Lübeck, Germany; 5 Department of Medicine, Karolinska Institute, Stockholm, Sweden; 6 Laboratory of Microbiology and Morphology of Tuberculosis, Institute of Phthisiopneumology “Chiril Draganiuc”, Chisinau, Republic of Moldova; 7 Autoimmun Diagnostika GmbH, Straßberg, Germany; 8 Division of Immune Cell-Analytics, Research Center Borstel, Borstel, Germany; University of Cape Town, SOUTH AFRICA

## Abstract

**Background:**

To evaluate interleukin (IL)-2 and interferon (IFN)-γ secreting T-cells in parallel for the differentiation of latent infection with *Mycobacterium tuberculosis* infection (LTBI) from active tuberculosis.

**Methods:**

Following ex-vivo stimulation of peripheral blood mononuclear cells (PBMC) with *M*. *tuberculosis*-specific antigens early secretory antigenic target (ESAT)-6 and culture filtrate protein (CFP)-10, immune responses were assessed by enzyme-linked immunospot IFN-γ release assay (EliSpot-IGRA) and a novel dual cytokine detecting fluorescence-linked immunospot (FluoroSpot) in 18 patients with pulmonary tuberculosis, 10 persons with previously cured tuberculosis, 25 individuals with LTBI and 16 healthy controls.

**Results:**

Correlation of IFN- γ^+^ spot-forming cells in EliSpot-IGRA and FluoroSpot were R^2^ = 0.67 for ESAT-6 and R^2^ = 0.73 for CFP-10. The number of IL-2^-^ IFN- γ^+^ producing cells was higher in patients with tuberculosis compared with past tuberculosis (CFP-10-induced p = 0.0068) or individuals with LTBI (ESAT-6-induced p = 0.0136). A cutoff value of >16 CFP-10-induced IFN-γ^+^ secreting cells/200.000 PBMC in the EliSpot-IGRA discriminated with highest sensitivity and specificity (89% and 76%, respectively). However, overlap in cytokine responses precludes distinction between the cohorts on an individual basis.

**Conclusions:**

Combined analysis of IFN-γ and IL-2 secretion by antigen specific T-cells does not allow a reliable differentiation between different states of *M*. *tuberculosis* infection in clinical practice.

## Introduction

Tuberculosis ranks among the leading causes of morbidity and mortality worldwide [1]. The World Health Organization (WHO) targets to eliminate tuberculosis as a public health problem by the year 2050 [2]. Unfortunately, lack of efficient screening tests for the identification of persons latently infected with *Mycobacterium tuberculosis* (LTBI), the delay in the diagnosis of active disease and the emergence of drug resistant strains of *M*. *tuberculosis* [3] are among the causes that jeopardize the achievement of the goal to eliminate tuberculosis in the near future. The most accessible tool for the diagnosis of LTBI is the tuberculin skin test, which has a very low specificity [4], and does not differentiate between *M*. *tuberculosis* sensitization and non-tuberculous mycobacterial (NTM) infections or history of *M*. *bovis* Bacille Calmette Guérin (BCG)-vaccination. Interferon-γ release assays (IGRA) represent an alternative to tuberculin skin tests and have emerged during the last decade as reference diagnostics for LTBI [5]. IGRAs measure the interferon (IFN)-γ release after stimulation of blood cells with *M*. *tuberculosis*- specific antigens [6]. Despite the fact that this new tool can differentiate between *M*. *tuberculosis* and NTM infections or *M*. *bovis* BCG-vaccination, IGRAs still fail to distinguish between active tuberculosis and LTBI [7]. Following tuberculosis, positive IGRA test results can persist in the absence of active disease. Recent publications suggest that the accuracy in discriminating LTBI from active tuberculosis can be improved by parallel assessment of the secreting profile of T-cells for other cytokines, such as interleukin (IL)-2 and/or tumor necrosis factor (TNF)-α [8–12]. Nevertheless, the clinical usefulness of this approach still needs to be investigated and this has so far mainly been addressed using flow-cytometry [10, 12, 13] that represents the technical gold standard for multiparameter analysis of immune cells. However, flow cytometry analysis is expensive and labour-intensive. The two colour FluoroSpot (AID, Straßberg, Germany) is a novel Enzyme-linked-immuno-Spot (EliSpot) technology, which enables to simultaneously assesses individual cells that secret *M*. *tuberculosis*-induced cytokines IFN-γ and IL-2 [11]. The FluoroSpot is cheaper and easier to perform than flow cytometer analysis [12].

We evaluated whether the analysis of dual (IFN-γ and IL-2) cytokine profile analysis by FluoroSpot is superior to the EliSpot-IGRA technology to distinguish between different states of *M*. *tuberculosis* infection.

## Participants and Methods

### Study participants

Following written informed consent patients with suspected tuberculosis or a documented history of previous tuberculosis from the Medical Clinic of the Research Center Borstel, Germany, and healthy controls, were recruited between December 2011 and December 2013.

Seventy-one participants were Caucasian, one patient with tuberculosis and another study participant with former tuberculosis were of Asian origin. All patients were tested negative for infection with human immunodeficiency virus 1. At the time of analysis 2/18 patients with tuberculosis had been on anti-tuberculosis treatment for a short period of time (<7% of their whole treatment duration).

Twenty mL venous peripheral blood was obtained and tested for the *M*. *tuberculosis*-specific immune response by EliSpot-IGRA (T-Spot.TB, Oxford Immunotec, Abingdon (UK) and FluoroSpot (AID, Straßberg, Germany) test systems. The four different study groups were defined as follows: 1) The diagnosis of tuberculosis was based on a positive *M*. *tuberculosis* culture result or a positive *M*. *tuberculosis*-specific nucleic amplification assay from sputum or a bronchopulmonary specimen, irrespective of EliSpot-IGRA test result. 2) Past tuberculosis was defined as diagnosed, treated and cured tuberculosis. Treatment had to be finished one year before the inclusion date. Inclusion was independent of the EliSpot-IGRA result. If reactivation of tuberculosis was suspected at admission, clinical data, imaging and a negative culture result had to exclude active disease. 3) Individuals with LTBI were defined by a positive response in the EliSpot-IGRA in individuals without any signs of tuberculosis disease [14]. 4) Healthy controls were volunteers with no history or sign of tuberculosis and a negative EliSpot-IGRA test result.

### Ethics Statement

The study was approved by the Ethical Committee of the University of Lübeck, Germany (05–096 and 12–072A). Written informed consent was obtained from all study participants. Reporting follows the STARD guidelines.

### Detection of IL-2^+^ and IFN-γ^+^ secreting cells

The cytokine response was studied on peripheral blood mononuclear cells (PBMC) isolated by Ficoll Hypaque density gradient centrifugation (Biochrom, Berlin, Germany). Duplicates of 200.000 cells per well were cultured overnight in 200 μL RPMI 1640 (PAA Laboratoris GmbH, Pasching, Austria) enriched with 5% fetal bovine serum (PAA Laboratoris GmbH, Pasching, Austria) on precoated 96-well plates. The EliSpot-IGRA assay was performed on T-Spot.TB plates from Oxford Immunotec Ltd., Abingdon, UK, the FluoroSpot assay was performed on FluoroSpot plates from AID, Straßberg, Germany. As recommended by the manufacturer, in the FluoroSpotassay all cells were cultured with anti-CD28 (0.5 μg/mL, AID, Straßberg, Germany). Unstimulated PBMC were used as negative control, PBMC stimulated with anti-CD3 (10ng/mL, clone X35, Beckman Coulter GmbH, Krefeld, Germany) for the EliSpot-IGRA and FluoroSpot, additionally pokeweed mitogen (PWM, 5μg/mL, Biochrom AG, Berlin, Bielefeld) served as positive control for the FluoroSpot. In both, the EliSpot-IGRA and FluoroSpot, specific stimulation was performed with the *M*. *tuberculosis*-specific antigens early secreted antigenic target 6 kDA (ESAT-6) and culture filtrate protein 10 kDA (CFP-10), 50 μL each, in a ready to use format from Oxford Immunotec. After 18 hours of culture the washing and staining procedures were performed as previously described for FluoroSpot [11] or according to the manufacturer’s instructions for EliSpot-IGRA [15].

Both, EliSpot-IGRA and FluoroSpot spots were counted with the AID EliSpot reader and software. Cytokine producing cells were expressed as number of spot-forming cells (SFC). The background response of the negative control was always deducted from specifically stimulated wells.

EliSpot-IGRA assay results were considered positive if more than five net SFC were counted after ESAT-6 or CFP-10 stimulation, and if the total number of SFC after specific stimulation was at least twice the number of SFC in the negative control well. EliSpot-IGRA results were considered negative if they did not meet the definition for a positive result and if the number of SFC in the positive control well was more than 20 SFC after subtraction of the number of spots in the negative control well and had at least twice the number of spots of the negative control well. Results that did not meet the criteria of positive or negative were considered indeterminate [16].

For a correct interpretation of the FluoroSpot, the positive controls anti-CD3 or pokeweed mitogen (PWM) had to induce more than 50 SFC/well of IL-2^+^ and more than 50 SFC/well IFN-γ^+^ secreting cells after subtraction of the number of spots in the negative control well and at least twice the number of spots of the negative control well, otherwise the test was assessed as indeterminate [11]. Providing a valid positive control result, the net SFC counts after ESAT-6 or CFP-10 stimulation was used as a continuous variable for each cytokine.

The performance of the EliSpot-IGRA and FluoroSpot was assessed for the accuracy of differentiation between the three different states of *M*. *tuberculosis* infection (active disease, past tuberculosis, LTBI) and healthy controls with negative EliSpot-IGRA results and agreement of both tests results between them. Culture results in the case of tuberculosis, EliSpot-IGRA results in the case of LTBI, healthy controls or past tuberculosis were used as reference standard. The discriminatory performance of the FluoroSpot was decided comparing the total number of IFN-γ^+^ or IL-2^+^secreting cells, the number of double producing cells secretingIL-2^+^IFNγ^+^, and of those exclusively secreting only one cytokine (IL-2^+^ IFN-γ^-^ or IL-2^-^ IFNγ^+^).

### Statistical analysis

For comparative analysis between groups the Mann-Whitney U-test for nonparametric data was used. Statistical tests for paired data were performed by Wilcoxon Signed Rank test. A p-value of <0.05 was considered significant. Concordance between EliSpot-IGRA and FluoroSpot results were assessed using R^2^coefficient. Bland-Altman test was used to figure the agreement between EliSpot-IGRA and FluoroSpot, calculating the mean difference (FluoroSpot—Eli Spot-IGRA/69) and the 95% limits of agreement (average difference ± 1.96 standard deviation of the difference) [17]. Receiver operator characteristics (ROC) analyses were performed to establish cutoffs of ESAT-6 and CFP-10-reactive cells producing the different cytokine combinations. The cutoff with the highest diagnostic sensitivity and specificity was determined using Youden index statistics. All statistical analyses were performed using the sixth version of GraphPad Prism (GraphPad Software, La Jolla, California, USA).

## Results

**Fig 1:**
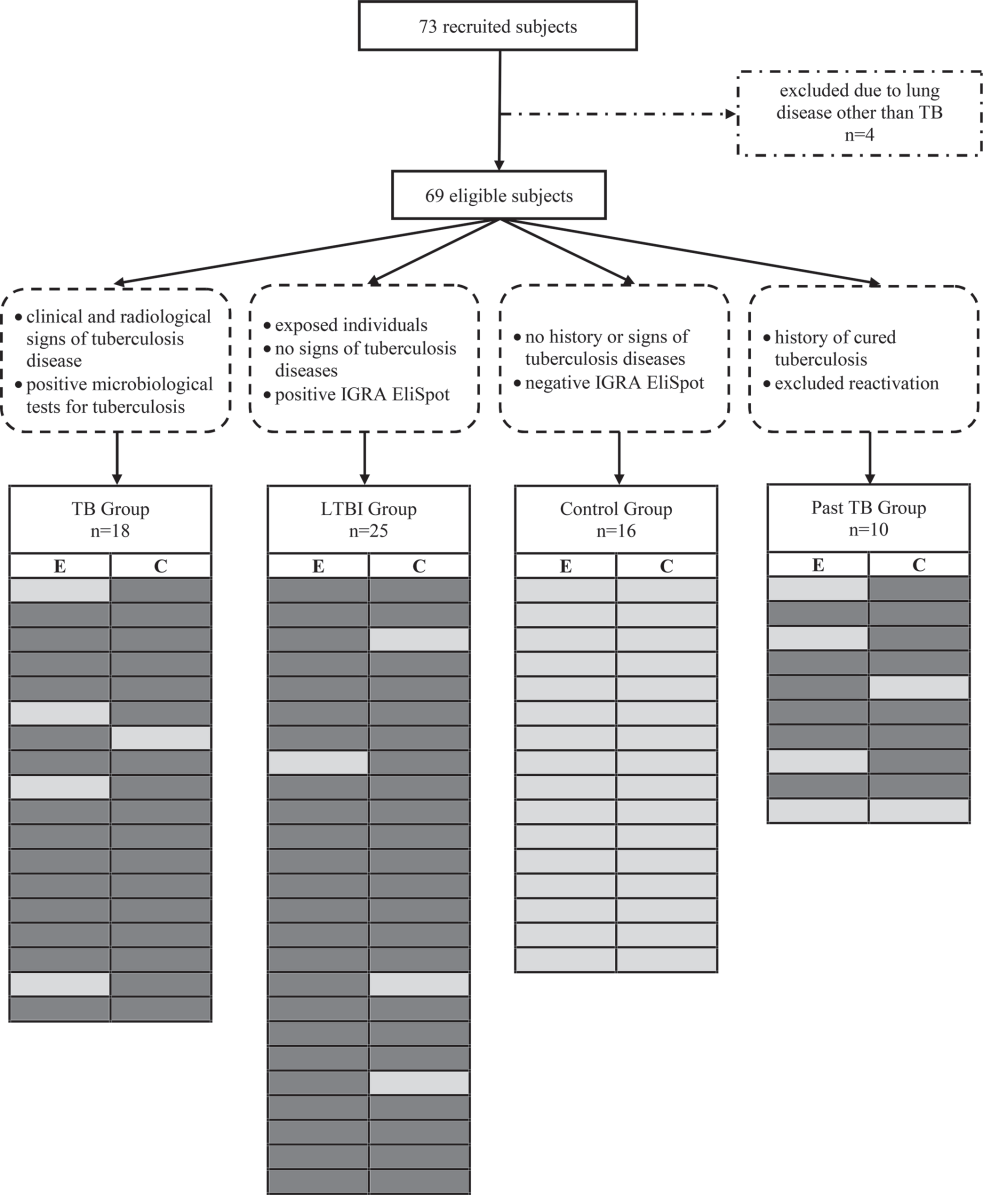
Flow chart of patients included in this study. TB = tuberculosis; LTBI = latent infection with *M*. *tuberculosis;* control = healthy individual with negative EliSpot-IGRA result; E = ESAT-6; C = CFP-10; dark grey = positive test result in the EliSpot-IGRA; light grey = negative test result in the EliSpot-IGRA.

A total of 73 recruited for this study. Four patients with suspected tuberculosis were excluded due to the final diagnosis a of lung disease other than tuberculosis (two patients with bronchial carcinoma, one pleuropneumonia, one disseminated pulmonary infection with *M*. *caprae*). The clinical characteristics of the 69 eligible participants and their corresponding EliSpot-IGRA results are depicted in Fig. 1, the demographical characteristics are shown in Table 1.

**Table 1:** Demographic characteristics of study subjects by groups.

	TB n = 18	Past TB n = 10	LTBI n = 25	Control n = 16	p-value
**Mean age ± standard error**	44,8±3,2	58,9±4,9	46,0±2,04	45,5±3,07	>0,05
**Gender m/f**	12/6	7/3	16/9	7/9	>0,05

TB = tuberculosis.

LTBI = latent infection with *M*. *tuberculosis*.

m = male.

f = female.

n = number of cases.

All 18 persons with active pulmonary tuberculosis had clinical and radiological findings consistent with tuberculosis and a positive EliSpot-IGRA immune response to at least one of the *M*. *tuberculosis*-specific antigens. Only one patient had a negative *M*. *tuberculosis* culture, but due to a positive nucleic acid amplification test and as both of his parents had positive cultures for *M*. *tuberculosis*, the patient was classified as a tuberculosis patient. In detail, 13 out of 18 patients with tuberculosis were positive by EliSpot-IGRA for both ESAT-6 and CFP-10, four patients reacted towards CFP-10 only and one patient was positive for ESAT-6 only (Fig. 1). Among the ten individuals with past tuberculosis, one had negative EliSpot-IGRA results for both ESAT-6 and CFP-10, whereas six individuals reacted towards ESAT-6 and eight individuals responded to CFP-10. In the LTBI group 24 out of 25 individuals had a positive ESAT-6 response and 22 a positive CFP-10 response. The 16 healthy controls had negative EliSpot-IGRA results.

**Fig 2:**
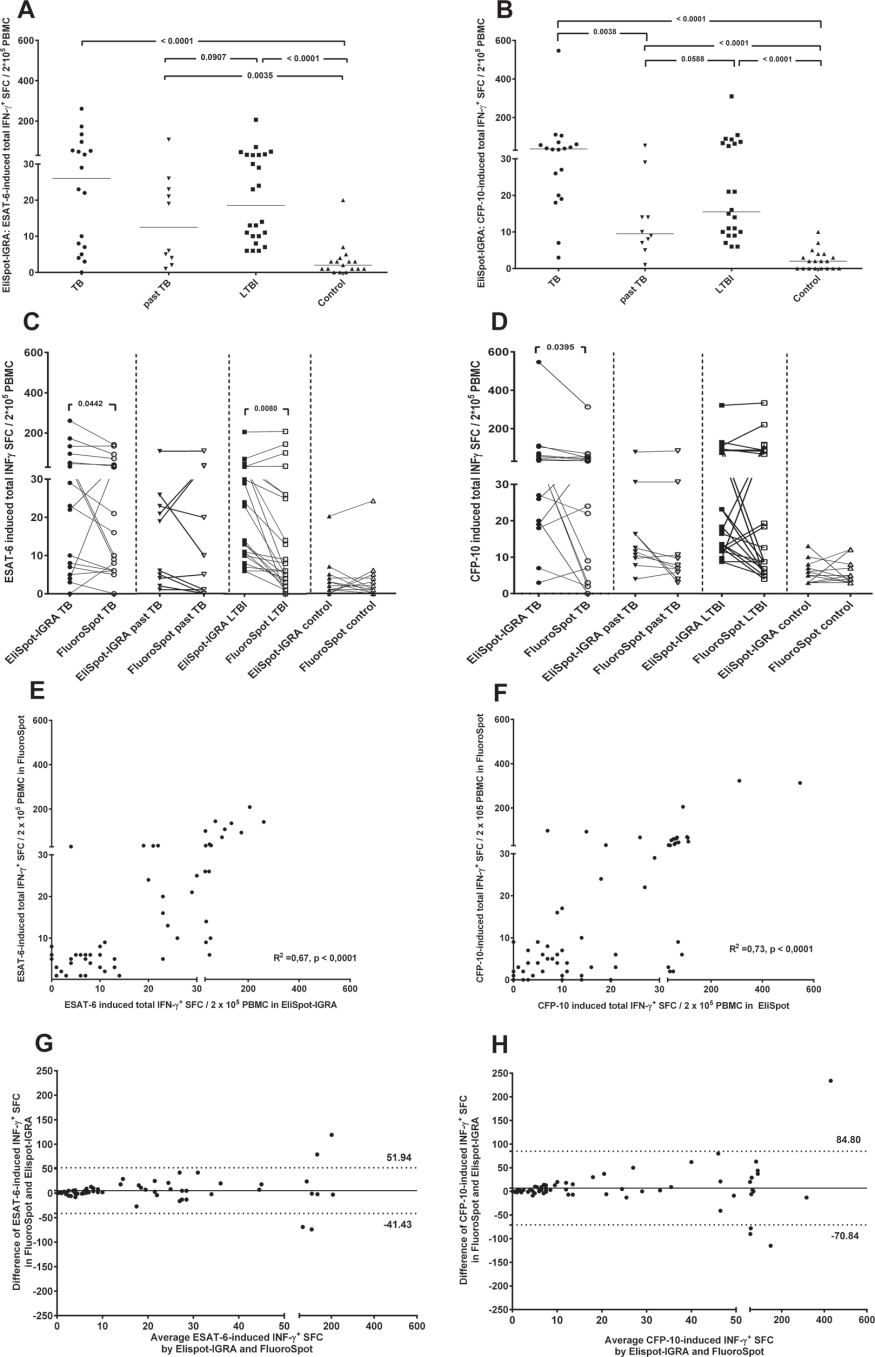
Detection of INF-γ^+^ in EliSpot-IGRA and their concordance with INF-γ^+^ results in FluoroSpot. ESAT-6 (A) and CFP-10 (B) -induced INF-γ^+^ immune response in 200.000 PBMCs/well in participants with active tuberculosis (TB, circle, n = 18), past tuberculosis (past TB, inverted triangle, n = 10), latent infection with M. tuberculosis (LTBI, square, ESAT-6-induced n = 24, CFP-10-induced = 22) and EliSpot-negative individuals (control, triangle, ESAT-6-induced n = 17, CFP-10-induced n = 19) was analysed. LTBI and controls had been defined according to their ESAT-6 and CFP-10-induced IFN-γ EliSpot-IGRA test result and clinical data. Number of INF-γ^+^ spot-forming cells (SFC) was enumerated by EliSpot. ESAT-6 (C) and CFP-10 (D) induced- INF-γ^+^ SFC in EliSpot-IGRA (solid symbols) and FluoroSpot (open symbols) were analysed as matched pairs (connected with lines), differences were calculated using Wilcoxon signed rank test. Correlation between the number of ESAT-6 (E) and CFP-10 (F) specific INF-γ^+^ spot-forming cells (SFC) in PBMC of 69 donors detected by FluoroSpot and EliSpot-IGRA. Concordance between EliSpot-IGRA and FluoroSpot results were assessed using R^2^ coefficient. Agreement by Bland–Altman test was expressed as mean difference (horizontal solid line) and 95% limits of agreement (dashed line) between ESAT-6 (G) and CFP-10 (H) induced- INF-γ^+^ SFC in FluoroSpot compared to EliSpot-IGRA.

As expected, neither ESAT-6 (Fig. 2A) nor CFP-10-induced IFN-γ response in the EliSpot-IGRA (Fig. 2B) allowed the discrimination between the different infection states of tuberculosis, past tuberculosis or LTBI.

### Concordance between EliSpot-IGRA and FluoroSpot assays results

To determine concordance between the two test assays, numbers of IFN-γ producing T-cells enumerated by the EliSpot-IGRA were matched with total IFN-γ response in the FluoroSpot assay of the same individual. Paired analysis of the IFN-γ^+^ SFCs of the 18 participants with tuberculosis were marginal different (ESAT-6 p = 0.0442, Fig. 2C; CFP-10 p = 0.0395, Fig. 2D), without any consequence for clinical practice, as all tuberculosis cases would have been detected by either one of the antigens in both tests. Detection of IFN-γ^+^ SFCs of participants with past tuberculosis or healthy controls did not reveal significant differences between the two test systems. Participants with LTBI showed significant lower ESAT-6- induced IFN-γ^+^ SFCs in FluoroSpot compared with the matched EliSpot-IGRA SFCs.

Correlation analysis of ESAT-6-induced and CFP-10-induced IFN-γ responses in EliSpot-IGRA and FluoroSpot (Fig. 2E-F) showed a moderate agreement of results (R^2^ = 0.67, p< 0,0001; R^2^ = 0.73, p< 0,0001, respectively).

Assessing the agreement between FluoroSpot and EliSpot-IGRA (Fig. 2G-H), FluoroSpot slightly underestimated the IFN-γ^+^ SFC detection. The mean difference of ESAT-6- induced IFN-γ^+^ was 5.254 SFCs (standard deviation SD 23.82) and 6.983 SFCs (SD 39.70) in CFP-10 induced IFN-γ^+^. EliSpot-IGRA and FluoroSpot results were widely distributed (ESAT-6 95% limits of agreement-41.43 to 51.94, CFP-10 -70.84 to 84.80), the variability of the differences increased as the magnitude of SFCs increased.

### Assessment of IL-2 and IFN-γ response induced by ESAT-6 in FluoroSpot assay

**Fig 3:**
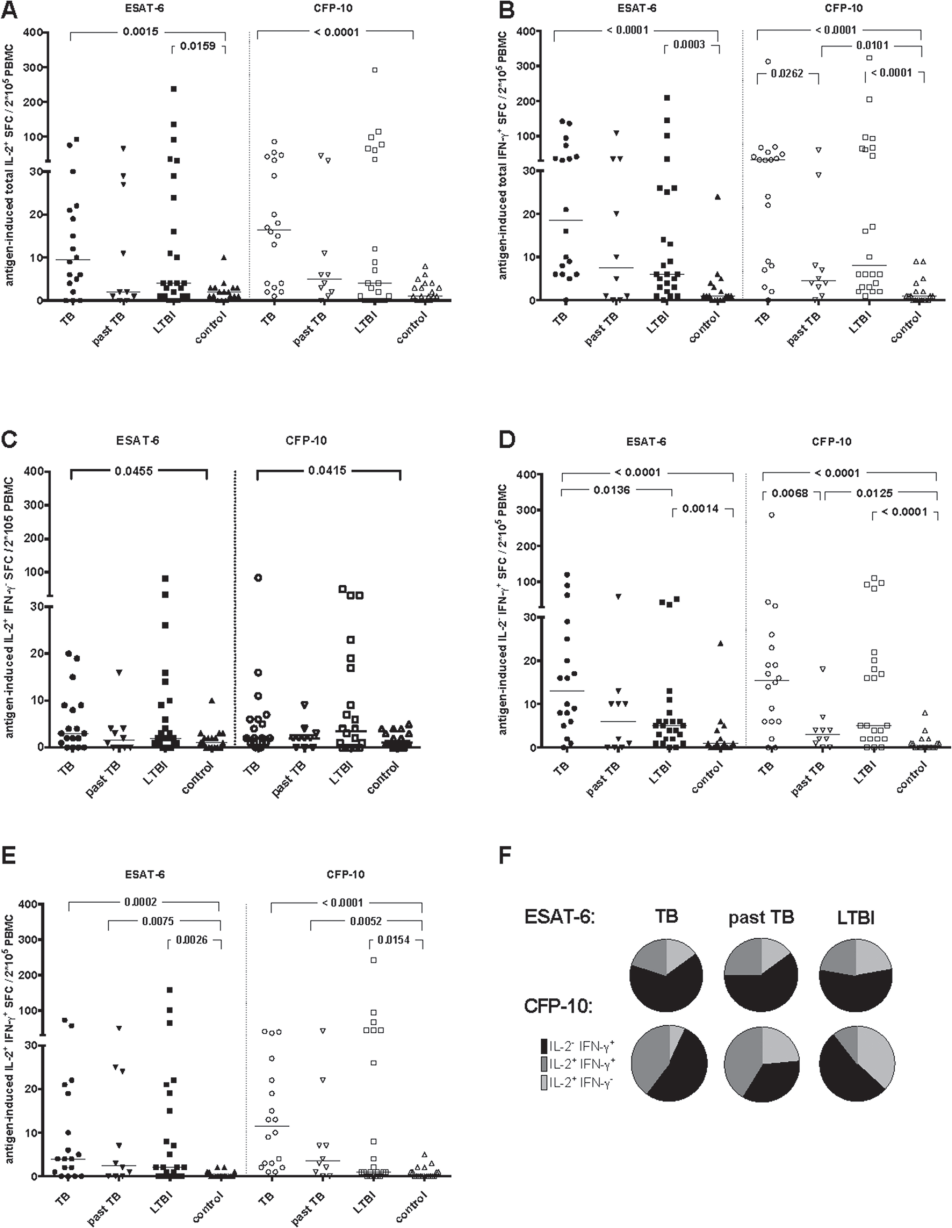
ESAT-6 and CFP-10- induced cytokine response in FluoroSpot. ESAT-6 induced cytokine immune response in 200.000 PBMCs/well in participants with active tuberculosis (TB, circle, n = 18), past tuberculosis (past TB, inverted triangle, n = 10), latent infection with *M*. *tuberculosis* (LTBI, square, ESAT-6-induced n = 24, CFP-10-induced n = 22), EliSpot-negative individuals (control, triangle, ESAT-6-induced n = 17, CFP-10-induced n = 19) was analysed. Groups had been defined according to the combination of their ESAT-6 and CFP-10 induced IFN-γ EliSpot-IGRA test result and clinical data. The number of IL-2^+^ (A), INF-γ^+^ (B), IL-2^+^ INF-γ^-^ (C), IL-2^-^ INF-γ^+^ (D) and IL-2^+^ INF-γ^+^ (E) spot-forming cells (SFC) were enumerated by FluoroSpot. (F) Mean proportion of ESAT-6 (top row) and CFP-10 (bottom row) -specific cytokine secreting cells for individuals with tuberculosis, past tuberculosis and LTBI are depicted as pie charts (light grey = IL-2^+^ INF-γ^-^, black = IL-2^-^ INF-γ^+^ and dark grey = IL-2^+^ INF-γ^+^ secreting cells). Mann-Whitney U-test for non-parametric data was used for comparative analysis. A p-value of <0.05 was considered significant.

Analysing the ESAT-6-induced cytokine response in the FluoroSpot system, the SFC of total IL-2^+^ secreting cells were significantly higher in tuberculosis or LTBI in comparison with the healthy EliSpot-IGRA-negative control group (median 9.5 SFC/200.000 PBMC versus 2 SFC/200.000 PBMC, p = 0.0015 and 4 SFC/ 200.000 PBMC versus 2 SFC/ 200.000 PBMC, p = 0.0159, respectively, Fig. 3A). Likewise, the number of total INF-γ^+^ producing cells differed significantly between active disease or LTBI in comparison with healthy EliSpot-IGRA-negative controls (median 18.5 SFC/ 200.000 PBMC versus 1 SFC/ 200.000 PBMC, p<0.0001 and 6 SFC/ 200.000 PBMC versus 1 SFC/ 200.000 PBMC, p = 0.0003, Fig. 3B). The only T-cell subset, which revealed significant differences between tuberculosis and LTBI were the ESAT-6-induced IL-2^-^ IFN-γ^+^ producing T-cells (median 13 SFC/ 200.000 PBMC versus 5 SFC/ 200.000 PBMC, p = 0.0136, Fig. 3D). In the same line individuals with tuberculosis trended to have higher frequency of IL-2^-^ IFN-γ^+^ producing T-cells in comparison to past tuberculosis (median 13 SFC/ 200.000 PBMC versus 6 SFC/ 200.000 PBMC, p = 0.095, Fig. 3D). Double cytokine producing T-cells IL-2^+^IFN-γ^+^ did distinguish between past tuberculosis in comparison with healthy EliSpot-IGRA-negative controls (median 2.5 SFC/ 200.000 PBMC versus 0 SFC/ 200.000 PBMC, p = 0.0075, Fig. 3E). Nevertheless the overlap in all five cytokine expression profiles did not allow clinical discrimination between the groups on an individual basis. Calculating the median proportion, distribution of IL-2^+^ IFN-γ^-^, IL-2^-^ IFN-γ^+^, and IL-2^+^ IFN-γ^+^ secreting T-cells in relation to the overall specific immune response did not differ between patients with tuberculosis or participants with past tuberculosis or LTBI (Fig. 3F).

### Assessment of IL-2 and IFN-γ response induced by CFP-10 using FluoroSpot assay

The analysis of the CFP-10-induced IL-2^-^ IFN-γ^+^ cytokine response in the FluoroSpot system did allow discrimination of past tuberculosis in comparison with active disease (median 3 SFC/ 200.000 PBMC versus 15.5 SFC/ 200.000 PBMC, p = 0.0068) or healthy EliSpot-IGRA-negative controls (median 3 SFC/ 200.000 PBMC versus 0 SFC/ 200.000 PBMC, p = 0.0125, Fig. 3D). The number of total INF-γ^+^, IL-2^+^ IFN-γ^+^ and IL-2^-^ IFN-γ^+^ was always significantly lower in healthy subjects in comparison with active tuberculosis, past tuberculosis and LTBI, as well as the SFC of total IL-2^+^ and IL-2^+^ IFN-γ^-^secreting cells of healthy EliSpot-IGRA-negative controls in comparison with tuberculosis patients. Nevertheless the median distribution cytokine profile (Fig. 3F) showed a dominance of IL-2^-^ IFN-γ^+^ in tuberculosis, in comparison to a balance between IL-2^+^ IFN-γ^-^, IL-2^+^ IFN-γ^+^ and IL-2^-^ IFN-γ^+^ in past tuberculosis and the highest proportion of IL-2^+^ IFN-γ^-^ in LTBI.

### Clinical utility of EliSpot-IGRA and FluoroSpot test systems

**Fig 4:**
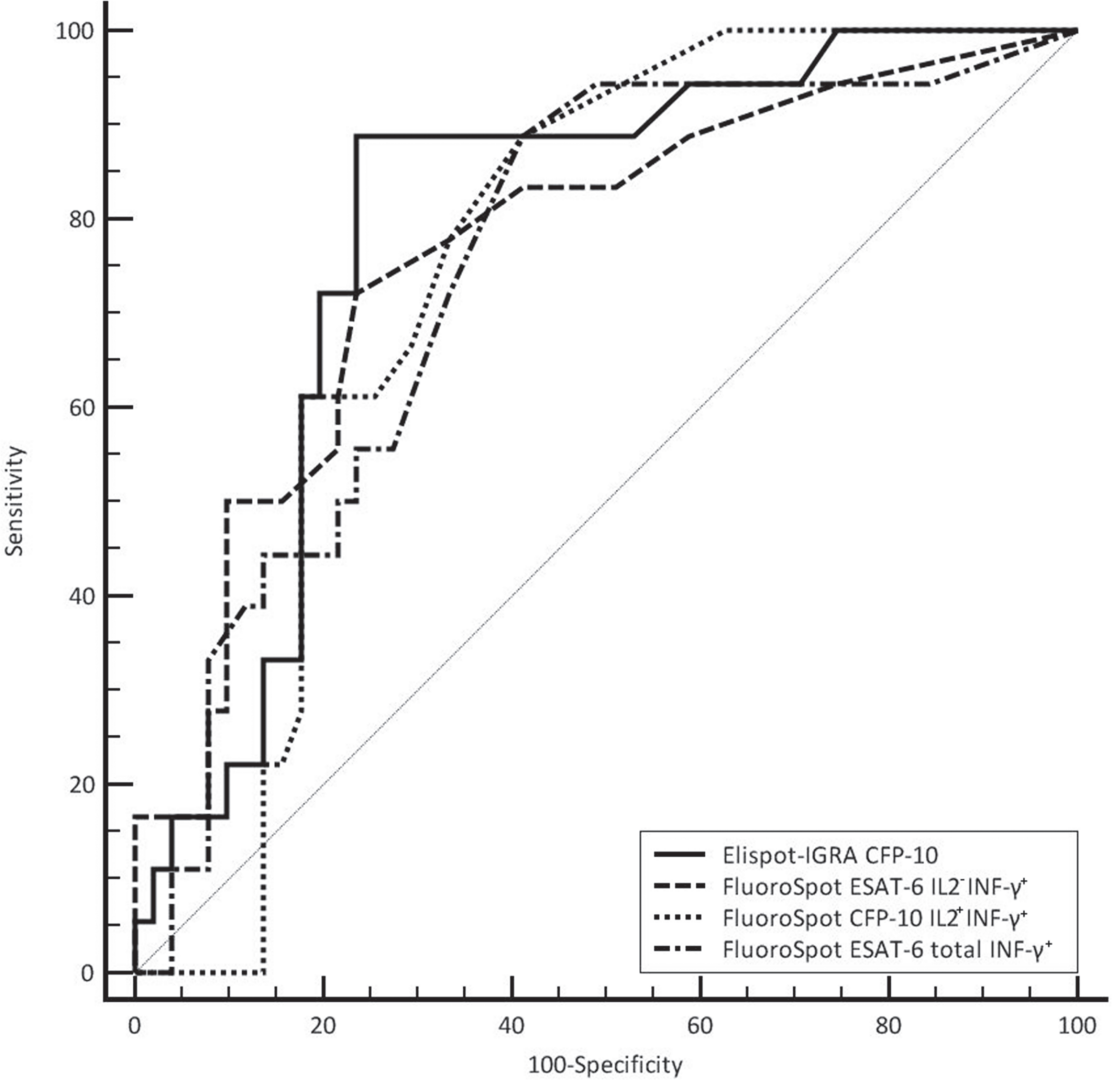
ROC plots of the FluoroSpot and EliSpot-IGRA tests with best discriminatory accuracy for subjects with active tuberculosis, assessed by AUC values.

The comparison of the receiver operator characteristics (ROC) analysis of EliSpot-IGRA and FluoroSpot revealed that the CFP-10-specific IFN-γ-response in EliSpot-IGRA performed as best discriminator between patients with active tuberculosis and persons with past TB, LTBI, or healthy controls (Fig. 4 and Table 2). At the optimum cutoff of > 16 SFC/ 200.000 PBMC of CFP-10-specific IFN-γ^+^ secreting cells of patients with active tuberculosis were identified with a specificity of 76% and sensitivity of 89% (AUC 0.795, 95% CI 0.680–0.882). Comparable results were achieved with the FluoroSpot cutoff of > 5 SFC/ 200.000 PBMC ofESAT-6-induced total IFN-γ^+^ secreting cells (AUC 0.751,95% CI 0.632–0.847, specificity 59%, sensitivity 89%) and > 6 SFC/ 200.000 PBMC of ESAT-6-induced IL-2^-^IFN-γ^+^ secreting cells (AUC 0.77, 95% CI 0.653–0.862, specificity 76%, sensitivity 72%). At the optimum cutoff of > 12 SFC/ 200.000 PBMC of CFP-10-induced total IL-2^+^secreting cells, the sensitivity and specificity for active tuberculosis were 82% and 67%, respectively (AUC 0.724, 95% CI 0.603–0.825). Thus, neither the analysis of IL-2^+^ nor IFN-γ^+^ producing cells allowed clear distinction between patients with active tuberculosis and LTBI or past tuberculosis on an individual level.

**Table 2:** Diagnostic performance of EliSpot-IGRA and FluoroSpot to differentiate between active tuberculosis and past tuberculosis/ LTBI.

			SFC cut off	Sensitivity % (95% CI)	Specificity % (95% CI)	positive LR (95% CI)	negative LR (95% CI)	AUC (95% CI)
**EliSpot-IGRA**	**ESAT-6**	**total IFN-γ^+^**	>33	44 (22–69)	88 (76–96)	3.78 (1.5–9.4)	0.63 (0.4–1.0)	0.68 (0.56–0.79)
	**CFP-10**	**total IFN-γ^+^**	>16	89 (65–99)	76 (63–87)	3.78 (2.2–6.4)	0.15 (0.04–0.5)	0.79 (0.68–0.88)
**Fluoro Spot**	**ESAT-6**	**total IL-2^+^**	>4	72 (47–90)	71 (56–83)	2.46 (1.5–4.1)	0.39 (0.2–0.8)	0.64 (0.52–0.75)
		**IL-2^+^ /IFN-γ^-^**	>3	44 (22–69)	75 (60–86)	1.74 (0.9–3.5)	0.75 (0.5–1.2)	0.59 (0.47–0.71)
		**total IFN-γ^+^**	>5	89 (65–99)	59 (44–72)	2.16 (1.5–3.1)	0.19 (0.05–07)	0.75 (0.63–0.84)
		**IL-2^-^/IFN-γ^+^**	>6	72 (47–90)	76 (63–87)	3.07 (1.7–5.4)	0.36 (0.2–0.8)	0.77 (0.65–0.86)
		**IL-2^+^ /IFN-γ^+^**	>3	61 (36–83)	73 (58–84)	2.23 (1.2–4.0)	0.54 (0.3–1.0)	0.64 (0.52–0.75)
	**CFP-10**	**total IL-2^+^**	>12	67 (41–87)	82 (69–92)	3.78 (1.9–7.4)	0.40 (0.2–0.8)	0.72 (0.60–0.82)
		**IL-2^+^ /IFN-γ^-^**	>0	89 (65–99)	31 (19–46)	1.3 (1.0–1.7)	0.35 (0.09–1.4)	0.59 (0.46–0.70)
		**total IFN-γ^+^**	>6	83 (59–96)	67 (52–79)	2.5 (1.6–3.9)	0.25 (0.09–0.7)	0.73 (0.61–0.83)
		**IL-2^-^/IFN-γ^+^**	>5	83 (59–96)	75 (60–86)	3.27 (2.0–5.5)	0.22 (0.08–0.6)	0.74 (0.63–0.84)
		**IL-2^+^ /IFN-γ^+^**	>1	89 (65–99)	59 (44–72)	2.16 (1.5–3.1)	0.19 (0.05–07)	0.75 (0.63–0.84)

LTBI = latent infection with *M*. *tuberculosis*.

ESAT-6 = early secreted antigenic target 6 kDA.

CFP-10 = culture filtrate protein 10 kDA.

IL-2 = Interleukin-2.

IFN-γ = Interferon gamma.

- = negative for cytokine production.

+ = positive for cytokine production.

CI = 95% confidence interval.

LR = likelihood ratio.

AUC = area under the curve.

## Discussion

This study was performed to evaluate the potential of the additional assessment of IL-2 secreting T-cells to IGRAs to overcome limitations of IGRAs in the differentiation of different states of *M*. *tuberculosis* infection. We found that cytokine profiles of T-cells towards ESAT-6 and CFP-10 differ significantly between the infection states of tuberculosis, past tuberculosis and LTBI. However, the inter-individual variability of cytokine profiles within one group of individuals hampers a clear discrimination between the groups. The highest sensitivity of 89% and specificity of 76% for the diagnosis of active tuberculosis was achieved from CFP-10-induced IFN-γ^+^ response in EliSpot-IGRA. Assessment of subpopulations of IL-2^+^ single, IL-2^+^ IFN-γ^+^ double, or IFN-γ^+^ single secreting cells in the FluoroSpot did not improve the differentiation between tuberculosis, past tuberculosis and LTBI.

The number of IFN-γ^+^ secreting cells in the two colour-based FluoroSpot correlated closely with EliSpot-IGRA results [12]. In contrast to previously published data [12] and despite the use of anti-CD28 as co-stimulatory antibody during the overnight cell culture in the FluoroSpot test system, FluoroSpot had very low background reactivity and SFC counts were lower than those obtained using EliSpot-IGRA. ROC analysis of the FluoroSpot SFC data revealed five and six SFC/ 200.000 PBMC for total IFN-γ^+^ and IL-2^-^ IFN-γ^+^ secreting cells respectively to be discriminative between active tuberculosis and past tuberculosis or LTBI. Though these calculated cutoffs are lower than the cutoff of ten SFC, which is recommended from the FluoroSpot manufacturer as cutoff for a positive immune response.

The FluoroSpot technique enables simultaneous detection of IL-2 and IFN-γ on a single cell level and thereby distinguishes between single secreting IL-2^+^ IFN-γ^-^ T-cells, double producing IL-2^+^ IFN-γ^+^ T-cells and single secreting IL-2^-^ IFN-γ^+^ T-cells, which are supposed to belong to different stages of T-cell differentiation, namely central memory T-cell, effector memory T-cell and terminally differentiated T-cells, respectively [18, 19]. The FluoroSpot detected significantly elevated ESAT-6-specific IL-2^-^ IFN-γ^+^ T-cells in tuberculosis in comparison to past tuberculosis and LTBI. This finding of an IL-2^-^ IFN-γ^+^ dominated profile in the case of tuberculosis disease goes along with that of others [11, 12, 20]. But nevertheless despite statistical significance, the overlap precludes distinction between the groups on an individual basis. In this study, ROC curve analysis revealed a cutoff of >16 SFC/ 200.000 PBMC CFP-10-induced IFN-γ^+^ secreting T-cells to be indicative for active tuberculosis with a sensitivity of 89% and a specificity of 76%. This means in clinical practice that <16 SFC/ 200.000 PBMC rules out active disease with good probability, but that the test is not accurate enough to rule in disease, as 24% of individuals with LTBI or past tuberculosis will get a false positive result being allocated to the diagnosis of tuberculosis. Differentiation of infection stages of *M*. *tuberculosis* by EliSpot-IGRA or FluoroSpot technology on PBMC is therefore still not feasible.

Previous findings on the dominance of IL-2^+^ IFN-γ^+^ and IL-2^+^ IFN-γ^-^ secreting cells in immune-controlled infection states of *M*. *tuberculosis*, e. g. during/after successful treatment, as well as in persons with LTBI [8, 11–13, 19, 21] are not confirmed by our findings. However, the results of cytokine profiling to distinguish between tuberculosis and LTBI are unequivocal [22, 23]. Some groups found that tuberculin purified protein derivative induced IL-2^+^ IFN-γ^+^ secreting cells effectively discriminate between active tuberculosis and non-active states, but—similar to our results- these differences were not observed for T-cells specific for ESAT-6 and CFP-10 [13]. Instead, others proved differences in ESAT-6/CFP-10-induced T-cell cytokine profiles between subjects with active and cured tuberculosis [8] and LTBI [12]. Discrepancy could have several reasons: different test assays (flow cytometry, ELISA-IGRA, EliSpot-IGRA, FluoroSpot), as well as different antigens preparations [18] and differences in protocols, e. g. prolonged time of incubation seems to account for differences due to increased cytokine secretion [19, 24, 25]. Disparities in study subjects’ characteristics might be caused by different exposure to mycobacteria in high or low incidence countries of tuberculosis as well as differences in bacterial load in disease; e.g. smear positivity was reported to decrease polyfunctional cytokine immune response [26]. Furthermore, the variation might just be due to inter-individual magnitude of immune responses. In order to adjust for this inter-individual variability of immune responses we have suggested to express individual cytokine response as proportion of the overall specific immune response of the individual values of IL-2^+^ IFN-γ^-^, IL-2^-^ IFN-γ^+^, IL-2^+^ IFN-γ^+^ responses to improve the differentiation of the *M*. *tuberculosis* infection states [11]. In our previous study a three-marker-model of the different cytokine-producing subpopulations, expressed as percentage of all cells producing these cytokines, has improved the discrimination of LTBI from active tuberculosis patients. In our present study this approach did not increase discrimination.

Our study has several limitations: The lack of IL-2 assessment by an IL-2 specific EliSpot-IGRA reduces the strength of the comparison between EliSpot-IGRA and FluoroSpot. In retrospect, the stimulation with tuberculin purified protein derivative antigen would have allowed broader comparison with results from other studies regarding differences between cytokine profiles of T-cells with specificity for ESAT-6/ CFP-10 and tuberculin [8, 13].

In conclusion, antigen-specific IL-2^-^ IFN-γ^+^ secreting T-cells are elevated in active tuberculosis in comparison to past tuberculosis and LTBI and can be easily identified by FluoroSpot. However, parallel diagnostic of IL-2 and IFN-γ secretion by antigen-specific T-cells does not allow a reliable differentiation between different states of *M*. *tuberculosis* infection in clinical practice.
